# Effect of Prolonged Discontinuation of L-Thyroxine Replacement in a Child with Congenital Hypothyroidism

**DOI:** 10.1155/2012/841947

**Published:** 2012-05-08

**Authors:** Rita Ann Kubicky, Evan Weiner, Bronwyn Carlson, Francesco De Luca

**Affiliations:** ^1^Section of Endocrinology and Diabetes, St. Christopher's Hospital for Children, Drexel University College of Medicine, Philadelphia, PA 19134, USA; ^2^Department of Pediatrics, Drexel University College of Medicine, Philadelphia, PA 19102, USA; ^3^Department of Emergency Medicine, St. Christopher's Hospital for Children, Drexel University College of Medicine, Philadelphia, PA 19134, USA

## Abstract

When diagnosed through neonatal screening and treated promptly and adequately, infants with congenital hypothyroidism (CH) experience normal physical growth and neurological development. Here we present a 3-year-old boy diagnosed with CH as a newborn, who was subsequently left untreated and experienced significant growth failure and developmental delay. This case emphasizes the importance of a consistent adherence to treatment in preventing such complications, especially in infancy and early childhood.

## 1. Introduction

The prevalence of congenital hypothyroidism (CH) in the past has been approximately 1 in 3,000 to 1 in 4,000 births with a higher incidence in Hispanic individuals [1]. More recent data suggest an increased prevalence of CH in the United States, possibly reflecting changes in screening methods [2, 3]. CH is the most frequent endocrine disease in infants, and, in most cases, the disorder is permanent [1]. When diagnosed and treated promptly and adequately, children born with CH experience normal physical growth and neurological development. Here we present a 3-year-old boy diagnosed at birth with congenital hypothyroidism, with subsequent poor compliance with L-thyroxine replacement.

## 2. Case Presentation

J. D. is a 2 11/12-year-old male, born in Mexico and diagnosed by neonatal screening with CH. He was then treated with L-thyroxine for approximately 1 year, until his mother lost her health insurance coverage and could not purchase the medication. Shortly after his family immigrated to the United States, J. D. was taken to St. Christopher's Hospital for Children's (SCHC) Emergency Department (ED) for evaluation.

**Figure 1:**
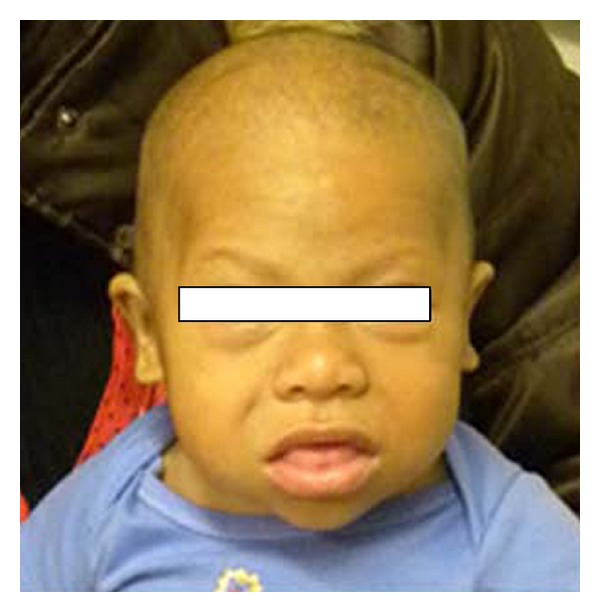
Photograph of patient demonstrating his coarse facial features and macroglossia.

**Figure 2:**
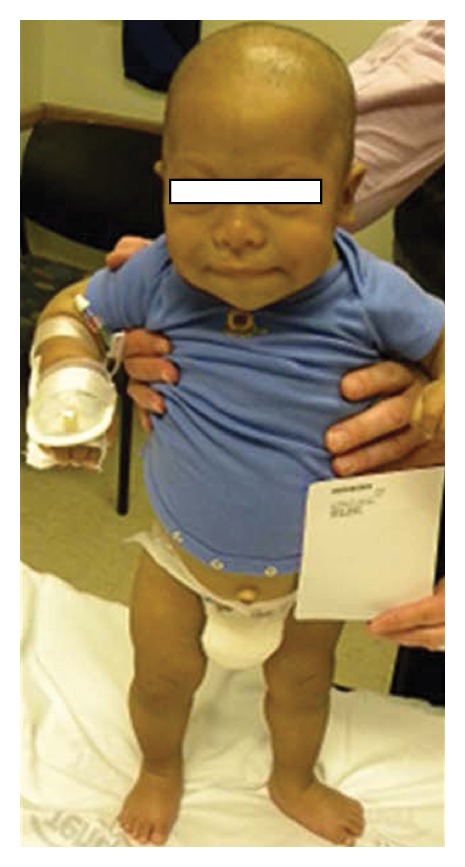
Patient stands with support. His height was significantly below the 3rd percentile (statural height of a 8 1/2-month-old male).

**Figure 3:**
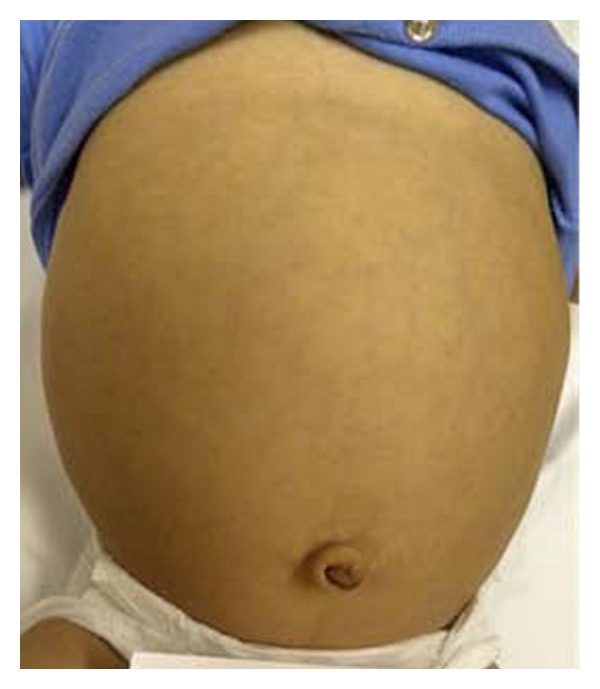
Protuberant abdomen and a small umbilical hernia.

His mother described a history of developmental delay, especially affecting his motor and language skills, as well as a history of constipation. Upon physical examination (PE), J. D.'s vital signs were as follows: temperature (*T*) of 97.6°F, heart rate (HR) of 95 beats per minute, blood pressure (BP) of 95/54 mmHg, and respiratory rate (RR) of 20 breaths per minute. His weight and length were both below the 3rd percentile (statural age of an 8 1/2-month old). J. D. was able to sit and crawl but could not stand or walk; he could only bear weight on his legs with support. The rest of his PE was significant for coarse facial features (Figures 1 and 2), macroglossia (Figure 1), a protuberant abdomen and a small umbilical hernia (Figure 3), and dry skin. His thyroid was not palpable. The serum TSH was very elevated (1,620 mIU/L) and the free T4 and thyroglobulin undetectable (Table 1); his serum prolactin level was mildly elevated (27.9 ng/mL). On the day of presentation, J. D. was placed on 50 *μ*g L-thyroxine daily (~5 *μ*g/kg/day), and a follow-up evaluation was arranged.

When evaluated at the SCHC Endocrinology Outpatient Clinic 2 weeks later, his mother reported increased activity, alertness, and appetite. In addition, his constipation had improved and he was less sleepy. His PE was virtually unchanged when compared to the one obtained in the ED. His dentition was delayed, with only 12 erupted teeth. His testicular volume was 4 cc bilaterally and he had no pubic hair.

A thyroid ultrasound revealed thyroid hypoplasia, with the right lobe measuring 5 mm anteriorly posteriorly (AP) and 6 mm in width (*W*), and the left lobe measuring 4 mm (AP) × 9 mm (*W*). Upon evaluation by a pediatric neurologist, his psychomotor developmental stage was found to be at the 18-month-old level for personal/social, fine motor/adaptive and language skills; his gross motor skills were at the 12-month-old level (by Denver's scale). His muscle tone was mildly but symmetrically decreased in all extremities, and his deep tendon reflexes were decreased.

J. D. was evaluated again at the Endocrinology Clinic 4 months after his initial visit to the ED. His mother reported that he had increased strength, which he was pulling to stand, trying to walk, and was much more active and alert. His testicular size was prepubertal. Laboratory studies revealed normal serum TSH and a free T4 (2.84 mIU/L and 1.5 ng/dL, resp.). Serum prolactin was also normal (Table 1).

After being treated with L-thyroxine for 7 months, J. D. grew 8 cm. He was enrolled in an early interventional program to improve his developmental skills. As per his mother, his vocabulary included more words and he was able to go up steps; he had also started to feed himself without help. Although his free T4 was normal (1.1 ng/dL), his TSH was found to be elevated (20.93 mIU/L).

J. D. was subsequently lost to followup for a few months because of change of insurance. Multiple attempts to contact his mother by phone and by certified mail were unsuccessful. He did not return to our clinic until 9 months later (16 months after his initial presentation): J. D. had grown 9.4 cm since his previous clinic visit (9 months prior). His maternal grandmother reported that he was a very active child who enjoyed singing and dancing. She described that his motor and language skills continued to improve: he was able to run, walk up and down stairs and he knew many words in both English and Spanish. His grandmother stated that his pediatrician at a local health center had been checking his thyroid function tests periodically. His grandmother also stated that J. D.'s dose of L-thyroxine had been increased to 75 mcg 2 months prior by his pediatrician; repeat thyroid function tests 1 month after the dose change were reported as normal. At our endocrinology clinic, thyroid function tests were obtained and a bone age study requested; a follow-up visit with his pediatric neurologist was also recommended. J. D.'s thyroid function tests revealed a normal TSH (0.79 mIU/L) and a borderline-high free T4 (1.7 ng/dL); as a result, the L-thyroxine dose was unchanged. His bone age, obtained one month after the last visit, was delayed (between 2 years 8 months and 3 years at the chronological age of 4 years and 5 months).

## 3. Discussion

Thyroid hormones play an essential role in normal statural growth: in fact, growth failure is a known complication of congenital hypothyroidism (CH). Before the neonatal screening was initiated in the 1970s, the percentage of children with CH having a height below the 10th percentile has been shown to range from 19% to 31% [4, 5]. After the introduction of the screening, several studies have reported a normal linear growth in infancy and childhood [6–8], while others have described a slight growth deceleration early in childhood in children with severe CH at diagnosis [9–11]. With respect to the achievement of a normal final height, some studies have suggested that the adequacy of L-thyroxine replacement in the first 6 months of life may influence the adult height of children with CH detected by newborn screening [12]. In contrast, other studies have found no correlation between severity at diagnosis, etiology, or initial L-thyroxine dosage [13, 14]. The only postnatal factor consistently found to be related to adult height has been the age at the start of treatment. Interestingly, longstanding untreated CH has been associated with permanent height loss. Boersma et al. have described two children with CH, who were left untreated for several years; although they experienced a marked catch-up growth, both of them reached an adult height below their target height [15].

It is well known that CH diagnosed on clinical grounds would often lead to severe cognitive and motor delays. Studies of children diagnosed in the prescreening era have reported that 8% to 29% patients affected by CH developed intellectual disability (i.e., IQ below 70) [5, 16, 17].

In contrast, a number of longitudinal studies of cohorts of children diagnosed with CH by screening have clearly demonstrated how hypothyroidism-related mental retardation can be successfully prevented [18–20]. On the other hand, while it is evident that early and adequate treatment can avoid serious sequelae, numerous studies have reported subtle but statistically significant neurological deficits in children with CH detected by screening when compared to unaffected individuals [21–23].

The initial severity of CH may be responsible for a less than ideal outcome. Indeed, evidence from multiple follow-up studies conducted in the United States and in Europe indicate that patients with lower T4 and more delayed skeletal maturation at the time of diagnosis by screening are more likely to exhibit subnormal cognitive skills [24–26]. Less obvious appears the effects of other variables: one study has shown that the timing of treatment initiation does not affect outcome [27], while other studies have demonstrated that the age at the start of treatment is correlated with neurological outcome in children with CH even after the introduction of the neonatal screening [18–21]. In addition, another study has indicated that higher initial L-thyroxine dose and faster time to normalization of thyroid function are important in preventing neurodevelopmental impairment [28].

Another variable thought to be an important prognostic factor for intellectual outcome is compliance to L-thyroxine treatment [19, 29, 30]. In a Norwegian study, neuropsychological tests administered at the mean age of 20 years to subjects with CH diagnosed by neonatal screening program have shown that their mean serum T4 level during the second year of life was a strong predictor of their verbal IQ [21]. In a German study of patients with CH with an early onset of treatment (9 days) and high initial L-thyroxine dose (14 *μ*g/kg/day), a decreased IQ score was correlated with poor compliance rather than with the severity of CH [31]. It appears that the discontinuation of L-thyroxine treatment experienced by our patient does not exclusively affect less affluent countries or families of low socioeconomic status. A retrospective analysis in the United States published in 2010 revealed that, by age of 3 years, the percentage of children with CH diagnosed by neonatal screening who had discontinued thyroxine replacement was 38%, and it was similar between Medicaid-enrolled and privately insured children [32]. While it is conceivable that some of these children were affected by transient hypothyroidism, many others may have been affected by permanent CH, like our patient, and thus are at risk for significant long-term sequelae especially if they are not treated adequately during the first three years of life until brain development is complete.

In conclusion, since neonatal screening for congenital hypothyroidism has been introduced, early L-thyroxine treatment has virtually eliminated hypothyroidism-related growth failure and severe neurological impairment. However, our patient demonstrates that prolonged lack of treatment due to poor compliance (despite early thyroid hormone replacement) may still be responsible for significant growth failure and psychomotor delay during childhood and possible permanent neurological deficits. This case emphasizes the importance of a consistent adherence to treatment in preventing such complications, especially in infancy and early childhood.

## Figures and Tables

**Table 1:** Initial and follow-up laboratory studies.

	Initial	6 weeks	4 months	7 months	16 months
TSH (mIU/L)	1620	5.2	2.84	20.93	0.79
Free T4 (ng/dL)^1^	<0.10 (<1.29)	1.41 (18.15)	1.5 (19.30)	1.1 (14.16)	1.7 (21.88)
Prolactin (ng/mL)^2^	27.9 (1,213.04)	10.1 (439.13)	5.1 (221.74)	—	—
Thyroglobulin (ng/mL)^3^	<0.5 (<0.5)	—	—	—	—

Values in Systeme International (SI) units are in parentheses.

^1^To convert to pmol/L multiply by 12.87.

^2^To convert to pmol/L multiply by 43.478.

^3^To convert to *μ*g/L multiply by 1.
